# Current progress of fluoroquinolones-increased risk of aortic aneurysm and dissection

**DOI:** 10.1186/s12872-021-02258-1

**Published:** 2021-09-28

**Authors:** Cui Jun, Bian Fang

**Affiliations:** 1 Department of Cardiothoracic Surgery, Xiangyang Central Hospital, Affiliated Hospital of Hubei University of Arts and Science, Xiangyang, 441000 Hubei China; 2 Department of Pharmacy, Featured Preparations of Vitiligo Xiangyang Key Laboratory, Xiangyang Central Hospital, Affiliated Hospital of Hubei University of Arts and Science, Xiangyang, 441000 Hubei China

**Keywords:** Aortic aneurysm, Aortic dissection, Fluoroquinolones, Adverse event

## Abstract

Aortic aneurysm (AA) and aortic dissection (AD) are major life-threatening diseases around the world. AA is a localized or diffuse dilation of the aorta, while AD is the separation of the layers creating a false lumen within the aortic wall. Fluoroquinolones (FQ) remain one of the most important kind of antibiotics and have a wider clinical use and broad antibacterial spectrum. FQ were also reported to treat infected AA. The most common adverse events (AEs) of FQ are mild and reversible, like headaches, diarrhea and nausea. Due to FQ-related serious AEs, such as tendonitis and tendon rupture, chondrotoxicity, or retinal detachment, QT-prolongation and dysglycemia, the United States Food and Drug Administration (FDA) issued a black box warning for FQ for systemic use in 2016 and updated warnings for FQ several times since then. Of note, in December 2018, FDA issued several “black box warnings” against FQ with the latest safety announcement warning about an increased risk of ruptures in the aorta blood vessel in certain patients. Recently, many studies have indicated an association between FQ and an increase risk of AA and AD. However, the exact mechanism of FQ-induced AA/AD remains unclear. This review aims to highlight the latest research progress of the alarming association between FQ and AA/AD. Moreover, molecular mechanisms of FQ in increasing risk of AA and AD are explored. Hopefully, this review can provide novel insights into FQ-increased the risk of AA/AD and a starting place for stewardship interventions.

## Introduction

Aortic aneurysm (AA) and aortic dissection (AD) are major life-threatening diseases worldwide. Large AA diameters are associated with increased risk of aneurysm rupture—AD, which is the most devastating complication of aortic diseases. AA is a localized or diffuse dilation of the aorta resulting in at lease a 50% increase in the aortic diameter, while AD is the separation of the layers creating a false lumen within the aortic wall [1]. Population-based studies reported an annual incidence of AA of 3–13.7/100,000 population, and AD of 3–20/100,000 population [2–4]. Notably, for the elderly population, the annual incidence of AA is much higher at 130/100,000 population [5]. Over the past decades, it is suggested that the rate of mortality from AA and AD has risen ranging from 1.2 to 24.8 fold in many developed countries, such as the United States, Britain and Japan [6, 7]. Accordingly to some researches, aortic aneurysm and dissection (AAD) is associated with the weakness of the aortic wall due to congenital defects, prolonged hypertension, or chronic inflammation [1]. In addition, other risk factors including smoking, old age, male sex (men have a fourfold increased risk compared to women) and family history of aortic disease are also implicated in AAD [8].

Fluoroquinolones (FQ) are the most widely used prescribed antibiotics in the world. In the United States alone, about 30 million outpatient prescribed with FQ per year [9]. FQ shows activity against both Gram-positive and Gram-negative bacteria, and are widely used to treat skin, urinary tract, bone, soft tissue, pulmonary infections and so on. Of note, FQ are also used to treat infected AA [8, 10]. It is reported that some adverse events (AEs) associated with FQ include tendonitis and tendon rupture, chondrotoxicity, peripheral neuropathy or retinal detachment, QT-prolongation and dysglycemia [8, 11]. The United States Food and Drug Administration (FDA) issued a black box warning for FQ for systemic use in 2016 and updated warnings for FQ several times since then [8]. Recently, many studies have indicated an association between FQ and an increased risk of AAD [1, 4, 9, 12].

In light of the escalating concerns, the latest research progress of the alarming association between FQ and AAD are summarized. Moreover, molecular mechanisms of FQ in increasing risk of AAD are explored. Hopefully, this review can provide novel insights into FQ-induced the risk of AAD and a starting place for stewardship interventions.

## Fluoroquinolones (FQ)

To our knowledge, FQ remain one of the most important kind of antibiotics used at present around the world. FQ have a wider clinical use and broad antibacterial spectrum against Gram-positive, Gram-negative, aerobic and anaerobic organisms [13]. Additionally, FQ were reported to exert activity on *Mycobacterium tuberculosis*, which may shorten the duration of treatment and reduce the emergence of bacterial resistance [14]. Besides their typical antibacterial activity, FQ also exhibited diverse atypical biological profiles, like anti-Alzheimer activities [15], anti-tumor [16], anti-malarial [17] and anti-tubercular [18].

Based on their spectrum of activity and their pharmacokinetic profile, FQ are classified into 5 categories. First generation FQ include nalidixic acid, oxolinic acid, pipemidic acid and rosoxacin, which have activity against Gram-negative bacteria [19]. However, they have limited clinical uses because of their short serum half-lives and are only used in Gram-negative bacteria-induced urinary tract infections (UTI) [19]. Second generation FQ contain ciprofloxacin, norfloxacin, enoxacin, pefloxacin, lomefloxacin, nadifloxacin, rufloxacin, and ofloxacin, which have a much wider activity against Gram-negative bacteria including *Pseudomonas aeruginosa*, and good activity against Gram-positive bacteria. Furthermore, they have longer serum half-lives. They are commonly used in tissue-based diseases, like complicated bladder infections, UTI, sexually transmitted diseases, specific types of pneumonia, pyelonephritis and skin infections [19]. Third generation FQ contain levofloxacin, pazufloxacin, temafloxacin, tosufloxacin, sparfloxacin, grepafloxacin, and balofloxacin, which have a much wider activity against Gram-negative bacteria including *P. aeruginosa*, and good activity against Gram-positive bacteria and anaerobes [20]. Moreover, they have longer serum half-lives. Fourth generation FQ include prulifloxacin, trovafloxacin, alatrofloxacin, delafloxacin, clinafloxacin, besifloxacin, sitafloxacin, finafloxacin, gatifloxacin, gemifloxacin, and moxifloxacin, which have a much wider activity against Gram-positive bacteria, especially *Streptococcus pneumoniae* and *Enterobacteriaceae*, good activity against atypical bacteria, and variable activity against anaerobes [21]. In addition, they have longer serum half-lives. Of note, they are considered to act at both DNA gyrase and topoisomerase IV and slow down process of bacterial resistance [20]. Fifth generation FQ include levonadifloxacin, nemonoxacin (non-fluorinated quinolone antibiotic), and zabofloxacin, which are undergoing clinical trials. It is suggested that they have higher potency against Gram-positive bacteria with better MIC values and Gram-negative bacteria. Zabofloxacin is considered to be effective in reproductive tract infections and UTI [22].

The most common AEs of FQ are mild and reversible, like headaches, diarrhea and nausea. However, we should pay more attention to FQ-related serious AEs, such as tendinitis and tendon rupture, hepatic toxicity, *Clostridium difficile* infections, prolonged QT interval, torsades de pointes, phototoxicity, dysglycemia, acute renal failure and seizures [23, 24]. A systematic review, meta-analysis and network meta-analysis conducted by Matok et al*.* [25] revealed that there was a significant association between FQ exposure and an increased risk of arrhythmia (85% increase) and cardiovascular mortality (71% increase). Specifically, moxifloxacin was considered to be ranked with the highest probability for arrhythmia and cardiovascular mortality [25]. Of note, in December 2018, FDA issued several “black box warnings” against FQ with the latest safety announcement warning about an increased risk of ruptures in the aorta blood vessel in certain patients.

## AAD

According to anatomical location, AA can be classified into thoracic aortic aneurysm (TAA) and abdominal aortic aneurysm (AAA). TAA exhibits much stronger hereditary and occurs commonly at younger ages with no obvious correlation with gender [26]. For example, some of these familial TAA patients harbor established monogenic forms of disease, like Marfan or Loeys-Dietz syndromes. However, it is reported that up to 20% of individuals with thoracic disease do not have a syndrome but do have related family members with disease (Familial Thoracic Aortic Aneurysm and Dissection or FTAAD) [27]. The risk factors associated with AAA include increasing age, male sex and lifestyle-related risk factors, like smoking, hypertension and hypercholesterolemia, which share many risk factors with atherosclerotic cardiovascular disease [28]. Additionally, inherited factors are also involved in the progression of AAA [29].

Numerous studies have highlighted the critical roles of smooth muscle function and extracellular matrix (ECM) (collagen and elastic fibers) in structural integrity of the arterial wall. Collagen and elastic fibers play important roles in the mechanical properties of the vessel wall. At the pathological level, TAA is characterized by medial degeneration, including elastic fibers fragmentation and loss of elastic tissue, vascular smooth muscle cells (VSMCs) loss, defects in collagen microarchitecture, proteoglycan deposition and a less remarkable inflammatory cells infiltration. Moreover, three important pathological marks are displayed in the progression of AAA: ECM degradation, VSMCs loss and infiltration of macrophages, neutrophils, mast cells and T/B lymphocytes. The imbalance between the production of active martix metalloproteins (MMPs) and their inhibitors (tissue inhibitor of MMPs, TIMPs) would cause the action active of MMPs to mediate ECM degration, which is implicated in the pathogensis of AA. According to the research, the expression of MMP-1, -9, -12, and -14 and MMP-2 were the most notably in the aortic wall of TAA, while MMP-1, -2, -3, -9, -12, and -13 were increased in AAA [30]. What is more, several studies described MMP-9 for TAA and MMP-3 for AAA as potentially important factors [30]. VSMCs in TAAD changed the phenotype from contractile to synthetic, and enriched with the expression of elastin- and collagen-degrading MMPs at the site of dissection [31]. In addition, lysyl oxidase (LOX) is a copper-dependent amine oxidase that catalyzes the formation of cross-linking in collagen fibers and elastic lamellae [27]. β-Aminopropionitrile (BAPN)-treated animals have been widely used as a model of AAD. As a lathyrogen, BAPN could irreversibly suppress the LOX, thus inhibiting the cross-linking of collagen fibers and elastic lamellae. Lee et al. [32] demonstrated that a missense mutation in LOX would cause AAD in humans.

## FQ-increased the risk of AAD

Recently, lots of laboratory-based studies and clinical studies have raised concern that FQ use could increase the incidence of AAD. Some recent systematic meta-analyses confirmed that there was a strong statistical association between FQ and AAD [12, 33–38]. By comparing with or without FQ exposure in 1 million general population in Taiwan, Lee et al. [1] found an increased risk in AAD with FQ use (rate ratio [RR] = 2.43; 95% confidence interval [CI] = 1.83–3.22). In the case-crossover study based on a large population-based database, Lee et al. [4] investigated twofold increase the risk of AAD after FQ exposure. In a self-controlled case series analysis, it is revealed that FQ should be used to serious infectious without appropriated alternatives because of its serious cardiovascular adverse events [39].

In a Canadian study, comparing with nonusers, Daneman et al. [40] suggested that FQ induced a similar increased risk of AA or AD. Otherwise, comparing with amoxicillin users, results in a Swedish study revealed that FQ increased a higher risk of AA but not AD [9]. Additionally, The latest study demonstrated that oral FQ were associated with increased incidence of aneurysm formation in United States adults (hazard ratio [HR] = 1.20; 95% CI 1.17–1.24) [41]. A meta-analysis revealed that the risk increase for FQ-induced AA alone was significant (adjusted RR = 2.23; 95% CI 2.01–2.45; I2 = 0%). However, the risk increase for FQ-induced AD alone was not significant (adjusted RR = 1.88; 95% CI 0.11–3.65; I2 = 74%) [33].

### Duration of FQ use and the incidence of AAD

As depicted in Table 1, susceptible period analysis further revealed that current FQ use within 60 days was associated with the highest risk of AAD [1, 4, 9, 12, 33, 40]. Lee et al. [4] observed that there was an increased risk of AAD with prolonged FQ use (OR = 2.41 for 3- to 14-day exposure; OR = 2.83 for > 14-day exposure; 95% CI 1.83–3.2). More specifically, FQ use within 60 days was associated with the highest risk of AAD.

**Table 1:** Duration of FQ use and the incidence of AAD

Duration of FQ use	No. of participants	OR (95% CI)	outcome	Reference	Study design
≤ 60	1477 case patients and 147,700 matched control cases	RR = 2.43, 95% CI 1.83–3.22	AA or AD	1	A nested case–control analysis
3–14	1213 hospitalized AA/AD patients	OR = 2.41; 95% CI 1.13–3.71		4	A case-crossover design
≤ 60	360,088	HR = 1.66; 95% CI 1.12–2.46	AA or AD	9	Nationwide historical cohort study
	1,744,360	Adjusted HR = 2.24, 95% CI 2.02–2.49	AA	40	Population-based longitudinal cohort study
		RR = 2.14; 95% CI 1.93–2.36	AA or AD	33	A systematic review and meta-analysis
	19,207,552	OR = 2.20; 95% CI 1.92–2.52	AA or AD	12	Cohort and case–control studies
> 14		OR = 2.83, 95% CI 1.06–7.57	AAD	42	A prospective population-based study
14–60	1213 hospitalized AA/AD patients	OR = 2.83; 95% CI 1.13–3.71	AA or AD	4	A case-crossover design
61–120	360,088	HR = 0.67; 95% CI 0.40–1.11	None	9	Nationwide historical cohort study
90	27,827,254	HR = 1.31; 95% CI 1.25–1.37	AAA	41	Cohort study
	27,827,254	HR = 1.60; 95% CI 1.33–1.91	Iliac artery aneurysm	41	Cohort study
	27,827,254	HR = 1.58; 95% CI 1.39–1.79	Other AAA	41	Cohort study

*FQ* fluoroquinolones, *OR* odds ratio, *HR* hazard ratio, *RR* adjusted ration, *CI* confidence interval, *AA* aortic aneurysm, *AD* aortic dissection, *AAA* abdominal aortic aneurysm, *AAD* aortic aneurysm and dissection

Howard et al. [42] found higher risk of AAD was associated with FQ exposure for longer than 14 days (OR = 2.83, 95% CI 1.06–7.57). What is more, Pasternak et al. [9] observed that there was no increased risk of AAD with FQ exposure for more than 60 days (61–120 days) (HR = 0.67; 95% CI 0.40–1.11). However, recent results indicated that FQ were associated with increased 90-day incidence of AAA (HR = 1.31; 95% CI 1.25–1.37), iliac artery aneurysm (HR = 1.60; 95% CI 1.33–1.91), and other AAA (HR = 1.58; 95% CI 1.39–1.79) [41]. Of note, the investigator suggested that FQ should be used with caution in adults, not just in high-risk individuals [41].

### Types and dosage forms of FQ use and the incidence of AAD

As shown in Table 2, ciprofloxacin, levofloxacin, and moxifloxacin are the most commonly prescribed FQ class of antibiotics, which are identified by the FDA and the EMA as being strongly associated with AAD [43]. In an recent study, Dolladille et al. identified 103 FQ-related AAD reports and a total of 264,917 FQ-related reports. They observed that all FQ (OR = 1.57; 95% CI 1.12–2.09), ciprofloxacin (OR = 2.89; 95% CI 2.24–3.74), levofloxacin (OR = 2.89; 95% CI 2.24–3.74) and moxifloxacin (OR = 3.22; 95% CI 2.21–4.71) increased the incidence of AAD [44]. Otherwise, there was no significant association of ofloxacin with the risk of AAD (OR = 0.79; 95% CI 0.33–1.91) [44].

**Table 2:** Types of FQ use and the incidence of AAD

FQ	Outcome	References
All FQ	AAD	[44]
Ciprofloxacin	AAD	[44]
	AA	[45]
Levofloxacin	AAD	[8, 44–46]
	AA	[45]
Moxifloxacin	AAD, AA	[44, 45]
	AA	[45]
Ofloxacin	None	[44]
Enoxacin, fleroxacin, gemifloxacin, grepafloxacin, lomefloxacin, norfloxacin, pazufloxacin, pefloxacin, prulifloxacin, rufloxacin, sparfloxacin, temafloxacin, trovafloxacin	None	[46]
Oral FQ	AAD	[1, 4, 8, 9, 45, 47]
Oral FQ	AAA, iliac artery aneurysm and other AAA	[41]

*FQ* fluoroquinolones, *AA* aortic aneurysm, *AAD* aortic aneurysm and dissection

By assessing three FQ (ciprofloxacin, levofloxacin, moxifloxacin)-related AAD from data (2004–2016) mining of the United States Food and Drug Administration Adverse Event Reporting System (FAERS), Sun et al. [45] indicated that they were associated with AA and only levofloxacin was associated with AD. Moreover, the incidence of AA was much higher than AD [45]. A case/non-case study using Vigibase® (2009–2015) observed the highest risk of AAD with levofloxacin [8]. With the Vigibase® between 1972 and 2017, François Montastruc et al. found that only levofloxacin increased the incidence of AAD (OR = 3.25, 95% CI 1.83–4.23) [46]. Additionally, no case of AAD was reported with enoxacin, fleroxacin, gemifloxacin, grepafloxacin, lomefloxacin, norfloxacin, pazufloxacin, pefloxacin, prulifloxacin, rufloxacin, sparfloxacin, temafloxacin, trovafloxacin [46]. In a mouse model of AAD, ciprofloxacin increased the incidence of AAD (38 of 48 [79%]; *P* = 0.001; χ^2^ = 10.9), severe AAD (32 of 48 [67%]; *P* < 0.001; χ^2^ = 15.7), and rupture and premature death (7 of 48 [15%]; *P* = 0.01; χ^2^ = 6.0) [47].

Data from FDA adverse event reporting system confirmed that oral FQ was more likely to induce AAD than intravenous administration [45]. In addition, compared with intravenous administration of FQ, oral FQ were more likely to be implicated in the increased risk of AAD [8]. In the case-crossover study based on a large population-based database, Lee et al. [4] found that oral FQ increased the risk of AAD (RR = 2.28; 95% CI 1.67–3.13). Consistent with this findings, a nationwide historical cohort study in Sweden (2006–2013) also demonstrated that oral FQ were associated with a 66% increased rate of AAD [9]. Lee et al. [1] conducted a nested case–control analysis of 1477 case patients and 147,700 matched control cases from Taiwan’s National Health Insurance Research Database (NHIRD) from among 1 million individuals longitudinally (2000.1–2011.11), the risk of AAD was increased in patients using oral FQ. Compared with amoxicillin-clavulanate, azithromycin, cephalexin, clindamycin, and sulfamethoxazole-trimethoprim, oral FQ can increase 90-day incidence of AAA (HR = 1.31; 95% CI 1.25–1.37), iliac artery aneurysm (HR = 1.60; 95% CI 1.33–1.91), and other AAA (HR = 1.58; 95% CI 1.39–1.79) [41]. A recent study confirmed that ciprofloxacin significantly increased the incidence of AAD (79%) in mice [47].

### Users of FQ and incidence of AAD

As depicted in Table 3, nationwide cohort study of adults with pneumonia or UTI results found that an increased relative rate of AA/AD associated with FQ within the pneumonia but not within the UTI [48]. Of note, FQ had an increased risk in AA/AD comparing to azithromycin (HR = 2.57; 95% CI 1.36–4.86; incidence, 0.03% for FQ vs 0.01% for azithromycin) but no increased rate comparing to combined trimethoprim and sulfamethoxazole (HR = 0.99; 95% CI 0.62–1.57; incidence, < 0.01% in both UTI groups). A recent reported revealed that FQ in patients with AD or AA had a higher risk of all-cause death (adjusted HR = 1.61; 95% CI 1.50–1.73), aortic death (adjusted HR = 1.80; 95% CI 1.50–2.15), and later aortic surgery [49].

**Table 3:** Users of FQ and incidence of AAD

OR (95% CI)	Outcome	Comorbid illnesses	Patient characteristic	References
HR = 2.57; 95% CI 1.36–4.86	AAD	Pneumonia		[48]
OR = 1.01; 95% CI 0.82–1.24	FQ didn’t increase the risk of AAD	Combined amoxicillin-clavulanate or ampicillin-sulbactam		[52]
OR = 0.88; 95% CI 0.70–1.11	FQ didn’t increase the risk of AAD	Combined with extended-spectrum cephalosporins		[52]
Adjusted HR = 2.24; 95% CI 2.02–2.49	AA		Older adults turning 65 years	[40]
HR = 1.66; 95% CI 1.12–2.46	AAD		Aged 50 years or older	[9]
RR = 1.72; 95% CI 1.37–2.16	AAD		Older than 70 years	[1]
HR = 1.18; 95% CI 1.09–1.28	AA		Aged 35 years or older (35–49 years)	[41]
HR = 1.24; 95% CI 1.19–1.28	AA		Aged 35 years or older (50–64 years)	[41]
RR = 1.83; 95% CI 1.27–2.64	AAD		Female patients	[1]
RR = 1.87; 95%CI 1.24–2.51	AAD		Female patients	[33]
OR = 0.92; 95% CI 0.46–1.86	Intracranial aneurysm or dissection			[52]
Adjusted HR = 1.61; 95% CI 1.50–1.73	All-cause death	AA or AD		[49]
Adjusted HR = 1.80; 95% CI 1.50–2.15	Aortic death	AA or AD		[49]

*FQ* fluoroquinolones, *OR* odds ratio, *HR* hazard ratio, *RR* adjusted ration, *CI* confidence interval, *AA* aortic aneurysm, *AD* aortic dissection, *AAD* aortic aneurysm and dissection

By analyzing the hospital admissions data containing information on 22 million adult hospitalizations in the United States from the Advisory Board billing and administrative database (2009–2015), researchers demonstrated that 18% of the 437,045 patients with AD were exposed to FQ and 19% of 27,876 AAD patients received FQ before the aortic repair. In addition, of the 1872 patients with Marfan syndrome, 14% received FQ during a hospital admission [8]. What’s more, Guzzardi et al. [50] suggested that patients with alpha-1 antitrypsin (A1AT) deficiency and longstanding FQ use (26 months) may have a higher risk of AAD.

Daneman et al. [40] observed a two- to threefold increase risk of AA in a larger longitudinal cohort study consisting of more than 1.7 million older adults turning 65 years in Ontario (HR = 2.72; 95% CI 2.53–2.93; adjusted HR = 2.24; 95% CI 2.02–2.49). Pasternak et al. [9] also described about twofold increase in AAD in 2.3 million elderly patients ( aged 50 years or older) with FQ use comparing to amoxicillin use (HR = 1.66; 95% CI 1.12–2.46). Lee et al. [1] found that the risk increase of AAD was more substantial in patients older than 70 years (RR = 1.72; 95% CI 1.37–2.16) than in patients 70 years or younger (RR = 1.46; 95% CI 0.98–2.18). And, it was more substantial in female patients (RR = 1.83; 95% CI 1.27–2.64) than in male patients (RR = 1.61; 95% CI 1.28–2.03) [1]. A meta-analysis revealed that FQ-increased the risk of AAD was higher in females compared to males (RR = 1.87; 95% CI 1.24–2.51; I2 = 0% versus RR = 1.58; 95% CI 1.25–1.92; I2 = 0%, respectively). What is more, it was higher in older patients compared to younger patients (RR = 1.72; 95% CI 1.3–2.07; I2 = 0% versus RR = 1.47; 95% CI 0.91–2.04; I2 = 0%, respectively) [33]. Otherwise, in a mouse model of AAD, they observed an increased risk of AAD in different aortic segments and no difference between male and female mice [47]. The latest study demonstrated that oral FQ were associated with increased incidence of aneurysm formation in United States adults (HR = 1.20; 95% CI 1.17–1.24) [41]. More specifically, adults aged 35 years or older had an increased risk in AA (18–34 years: HR = 0.99; 95% CI 0.83–1.18; 35–49 years: HR = 1.18; 95% CI 1.09–1.28; 50–64 years: HR = 1.24; 95% CI 1.19–1.28; *P* = 0.04) [41].

Of course, there are different points of view in some studies. Relative to combined amoxicillin-clavulanate or combined ampicillin-sulbactam (OR = 1.01; 95% CI 0.82–1.24) or with extended-spectrum cephalosporins (OR = 0.88; 95% CI 0.70–1.11) among patients with indicated infections, Dong et al. [51] observed that FQ didn’t increase the risk of AAD in a nested case–control study. A case-time-control study was performed with French National Insurance databases covering > 60 million inhabitants. Comparing with amoxicillin (OR = 0.97; 95% CI 0.61–1.53), there was no an excess of risk of intracranial aneurysm or dissection with FQ exposure (OR = 0.92; 95% CI 0.46–1.86) [52].

### Genetic predisposition and FQ-induced increased risk of AAD

According to some finds, there are multiple genetic syndromes associated with aortic aneurysmal disease, such as Marfan syndrome (*FBN1*), Ehlers-Danlos syndrome (*COL5A1*, *COL5A2*, *COL3A1*), Loeys-Dietz syndrome (*TGFBR1*, *TGFBR2*), FTAAD (*TGFBR2*, *MYH1*, *ACTA2*), and autosomal dominant polycystic kidney disease (*PDK1*, P*KD2*) [53]. Genetic studies explored that ciprofloxacin-associated neuropsychiatric toxicities may related to one specific *CYP450* gene in patients [54]. Of note, Guzzardi et al. [50] suggested that patients with alpha-1 antitrypsin (A1AT) deficiency and longstanding FQ use (26 months) may have a higher risk of AAD. However, there are a few reports about genetic predisposition and FQ-induced increased risk of AAD.

## Mechanisms of FQ-induced AAD

**Fig. 1:**
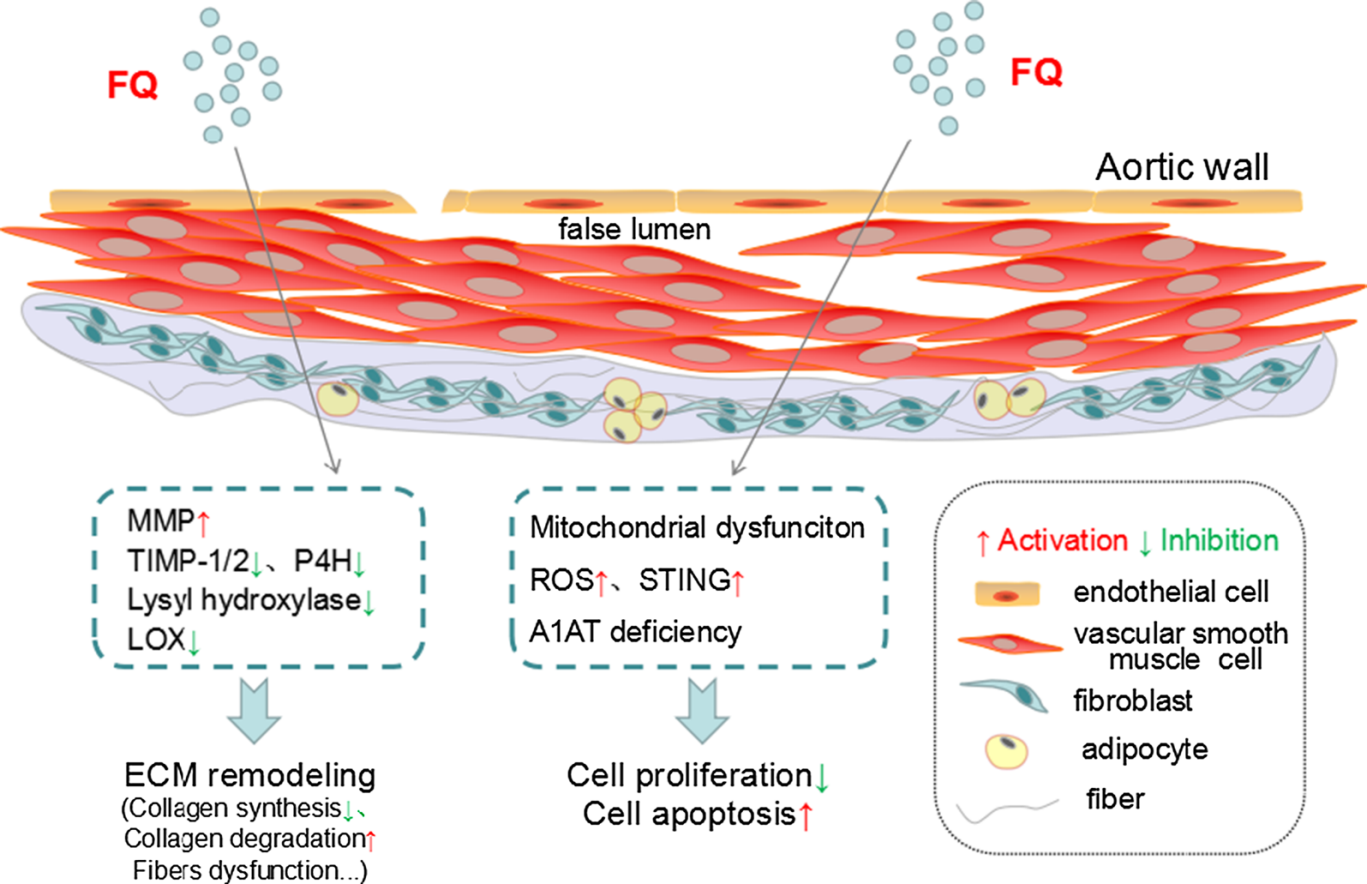
Mechanisms of FQ-induced AAD. FQ induces ECM remodeling via promoting MMP activation and inhibiting TIMP-1/2, P4H, Lysyl hydroxylase and LOX. FQ decreases cell proliferation and increases cell apoptosis through promoting mitochondrial dysfunction, ROS production, activation of STING. Patients with A1AT deficiency may associated with FQ-induced AAD. *FQ* fluoroquinolones, *MMP* martix metalloprotein, *TIMP* tissue inhibitors of matrix metalloproteinase, *P4H* prolyl 4-hydroxylase, *LOX* lysyl oxidase, *ROS* reactive oxygen species, *STING* stimulator of interferon genes, *A1AT* alpha-1 antitrypsin, *ECM* extracellular matrix, *AAD* aortic aneurysm and dissection

The exact mechanism of FQ-induced AAD remains unclear. Some hypothesized biological mechanisms have been proposed, as depicted in Fig. 1.

FQ may interfere with ECM integrity in the aortas. Dysregulation of ECM homeostasis disrupted ECM integrity and impaired biomechanical strength, which finally triggered progressive aortic weakening, dissection, or rupture [55]. According to some studies, enzymatic degradation of ECM by MMPs and vascular remodeling constituted the most prominent characters of AA [56]. However, TIMPs may inhibit the development of AA. Evidence confirmed that FQ reduced collagen production in tenocytes [57] and fibroblasts [58]. Plenty of researches showed that ciprofloxacin (the most commonly used FQ) suppressed TIMP-1 expression but enhanced MMP expression in the cornea [59], in tendon cells and tissues [57, 60], and in fibroblasts [61], which finally promoted MMP activation and tissue destruction.

It was suggested that ciprofloxacin greatly up-regulated MMP activity more than twofold in cultured human aortic smooth muscle cells (HASMCs) [62]. Human aortic fibroblasts exposed to FQ showed an increased capacity for ECM dysregulation by reducing the expression of collagen and endogenous protease inhibitors protein. They further demonstrated collagen degradation and decreased TIMPs activity in human aortic fibroblasts cultured with 2 days FQ [63]. A recent study confirmed that ciprofloxacin significantly increased the incidence of AAD (79%) in mice [47]. Specifically, in the mouse model of AAD that ciprofloxacin exposure reduced the expression of LOX, an critical enzyme in the assembly and stabilization of elastic fibers and collagen. Meantime, ciprofloxacin us enhanced MMP expression and activity as well as elastic fiber fragmentation in the aortas. Furthermore, in cultured smooth muscle cells, ciprofloxacin markedly down-regulated LOX levels and activity while up-regulated MMP levels.

Myofibroblasts have an abundant cell population in the aortic wall adventitia and have a capacity for structural arterial ECM remodeling. Another recent study found that FQ exposure induced an imbalance of ECM regulatory processes and enhance the capacity of human aortic myofibroblasts for ECM remodeling [64]. In addition, ciprofloxacin greatly reduced TIMP1 and TIMP2 expression while induced MMP9 to TIMP2 ration and collagen-1 expression in cultured human aortic myofibroblasts [64].

To our knowledge, FQ is considered to be powerful iron chelators. Prolyl 4-hydroxylase (P4H) and lysyl hydroxylase are iron-dependent enzymes, which play central roles in the post-translational modification of collagen. These enzymes promote collagen maturation through hydroxylation of proline and lysine residues to induce collagen cross-linking, which is essential for the tensile strength of collagen fibers. Badal et al. [65] demonstrated that FQ (norfloxacin, ciprofloxacin, enrofloxacin)-meidated iron chelation suppressed collagen maturation by inhibiting P4H and lysyl hydroxylase. FQ also exerted chelating properties against some other ions, including magnesium [66, 67] and calcium [67], which finally influenced collagen synthesis.

In addition, the effect of FQ on the inhibition of cell proliferation and induction of cell apoptosis may lead to aortic destruction. Evidence showed that FQ inhibited cell proliferation in various cells, including tenocytes [68], osteoblasts [69] and chondrocytes [70], and induced cell apoptosis in various cells, like tenocytes [57] and lens epithelial cells [71]. A recent study found that ciprofloxacin induced aortic cell injury in the aortas in a mouse model of AAD [47]. Furthermore, ciprofloxacin suppressed cell proliferation and promoted cell death in cultured HASMCs [47]. Mechanically, as a DNA topoisomerase inhibitor, ciprofloxacin induced nuclear and mitochondrial DNA damage and the the release of DNA, which finally promoted mitochondrial dysfunction, reactive oxygen species (ROS) production, activation of STING (stimulator of interferon genes, the cytosolic DNA sensor) and cell death [47]. However, in cultured human aortic myofibroblasts, ciprofloxacin exerted no significant effects on cell apoptosis, necrosis and metabolic viability [64].

## Conclusion and future prospects

According to the studies, exposure to FQ is substantially associated with the increase risk of AAD. Given the global burden of AAD and the growing FQ use around the world, well-designed studies in populations, especially high-risk populations, should be conducted. It is worthy to launch animal studies to clarify the pathophysiological mechanism of this association. What is more, it would be especially important to illustrate how FQ interact with the aortas. Better and direct understanding of the exact mechanism of FQ—induced AAD is imperative and essential. The last, but not the least, clinicians should carefully consider and balance the risks of AAD associated with FQ exposure against their established benefits.

## Data Availability

Not applicable.

## Abbreviations

AA: Aortic aneurysm
AD: Aortic dissection
FQ: Fluoroquinolone
AEs: Adverse events
FDA: Food and Drug Administration
TAA: Thoracic aortic aneurysm
AAA: Abdominal aortic aneurysm
FTAAD: Familial Thoracic Aortic Aneurysm and Dissection
ECM: Extracellular matrix
VSMCs: Vascular smooth muscle cells
MMPs: Martix metalloproteins
BAPN: β-Aminopropionitrile
FAERS: Food and Drug Administration Adverse Event Reporting System
HASMCs: Human aortic smooth muscle cells
